# Development of eye phantom for mimicking the deformation of the human cornea accompanied by intraocular pressure alterations

**DOI:** 10.1038/s41598-022-24948-2

**Published:** 2022-11-30

**Authors:** Han Saem Cho, Sae Chae Jeoung, Yun Sik Yang

**Affiliations:** 1 Advanced Instrumentation Institute, Korea Research Institute of Standards and Science, Daejeon, 34113 Korea; 2 Department of Ophthalmology, Wonkwang University School of Medicine, Iksan, 54538 Korea

**Keywords:** Health care, Materials science

## Abstract

Comparative studies between artificial eyeball phantoms and in-vivo human subjects were carried out to better understanding the structural deformation of the cornea under varying intraocular pressure (IOP). The IOP-induced deformation and the tension of the cornea were measured by using an optical coherence tomography and noncontact tonometer readings, respectively. The dependence of the central cornea thickness (CCT) and corneal radius of curvature (CRC) on the IOP differed significantly between the full eyeball phantom (FEP) and cornea eyeball phantom (CEP) models. While the CCT changes were very similar between the two models, the relation between the CRC and the IOP was dependent on the type of eye phantom. For the CEP, the CRC drastically decreased as internal pressure increased. However, we found that the changes in the CRC of FEP was dependent on initial CCT under zero IOP (CCT_0_). When CCT_0_ was less than 460 μm, the CRC slightly decreased as IOP increased. Meanwhile, the CRC increased as IOP increased if CCT_0_ was 570 μm. A constitutive mechanical model was proposed to describe the response of the cornea accompanied by the changes in IOP. In vivo measurements on human subjects under both noninvasive and invasive conditions revealed that the relation between the CRC on the IOP is much closer to those observed from FEP. Considering the observed structural deformation of human cornea, we found that FEP mimics the human eye more accurately than the CEP. In addition, the tonometry readings of IOP show that the values from the CEP were overestimated, while those from the FEP were not. For these reasons, we expect that the FEP could be suitable for the estimation of true IOP and allow performance testing of tonometers for medical checkups and other clinical uses.

## Introduction

After the introduction of a phantom in the shape of a medication cup to evaluate the performance of artificial silicon vitreous implants^1^, many different types of eye phantoms have been designed to reproduce the characteristics of the human eye to be used for various purposes: prosthetics, surgery simulation^2–4^, and testing and evaluation of diagnostic instruments^5–8^, such as optical coherence tomography (OCT) machines and tonometers. The shapes of eye phantoms vary according to their purposes. Intraocular pressure (IOP), which is defined as the fluid pressure inside the eyeball and usually measured by tonometry, plays an important role in diagnosing glaucoma and in monitoring its progression and therapeutic response. Almost all tonometers estimate IOP by measuring the force required to deform the cornea. It is invaluable to use an eye phantom to mimic as accurately as possible the structural deformation and the tension of the cornea accompanying a change in IOP in order to ensure a reliable measurement.

To fulfill the requirements of the international standard for performance testing on a tonometer (ISO 8612), at least 120 human subjects should be tested with no more than 5% of the paired differences (± 5 mmHg) between the reference tonometer readings and the test tonometer readings. It is time-consuming and rather expensive to conduct large-scale population studies on human subjects to confirm that a tonometer meets this standard. To solve this issue, it is important to develop eye phantoms whose structural deformation and tension can be measured from the outside upon the application of IOP and whose characteristics mimic the human eye fairly well. Because most applanation tonometers measure IOP readings based on the force needed to flatten a predetermined area of the corneal surface, this force is a function of the stiffness and shape of the cornea. Thus, it is difficult to estimate the true IOP from tonometer readings without a detailed understanding of the contribution of IOP-induced deformation in addition to that of the corneal thickness. Most eye phantoms used for evaluating the performance of tonometers are fixed-cornea eye phantoms. This type of eye phantom mimics the deformation of the human eyeball only to a certain extent.

The deformation of the cornea due to IOP changes is expected to be governed by its biomechanical properties. Additionally, the biomechanical properties of ocular tissues including the cornea, sclera, ciliary body and lens are expected to affect tonometry readings. Many studies^9–11^ on the biomechanical properties of eye tissues have shown that the tissues exhibit highly nonlinear stress–strain behavior as IOP increases. Using optical coherence elastography (OCE), Z. Han et al.^9^ reported an increase in the stiffness of enucleated porcine eyes after corneal collagen crosslinking treatment. J. J. Pitre et al.^10^ investigated the biomechanical properties of porcine corneas ex-vivo by using acoustic micro-tapping OCE and suggested that the biomechanical behavior of enucleated porcine corneas should be described with two different shear moduli. S. Park et al.^11^ investigated the relations between IOP and the biomechanical properties of the crystalline lens in enucleated bovine eyes by measuring the elastic waves propagating in the ocular tissues. A. Ramier et al.^12^ reported the shear modulus of 72 ± 14 kPa in human corneas in-vivo by using OCE.

Corneal features account for more than 60% of human vision. However, there is no precedent works on the relation between IOP alterations and the shape of the cornea. When combined with the complex anatomical structure of the ocular tissues, the nonlinearity in the biomechanical properties makes it difficult to achieve a deeper understanding of the detailed relations between IOP and the deformation of the cornea. Here, we report a novel method to manufacture full eyeball phantom (FEP) made of the soft and flexible materials of polydimethylsiloxane (PDMS), which has been used for pharmaceutical and medical applications such as a glaucoma drainage implants and artificial skin^13,14^. We also fabricated cornea eyeball phantom (CEP) and used them for comparative studies. We assessed the validity of these two types of eye phantoms for mimicking human subjects by measuring their structural deformation and noncontact tonometer (NCT) readings at varying IOPs. And then, we have carried out the comparative in-vivo measurements of corneal structural deformation of human subjects resulted from the IOP changes by both noninvasive and invasive method. In vivo measurements on human subjects clearly show that the dependence of the shape of the cornea on the IOP observed from human subjects is much closer to those observed from FEP.

## Results

**Figure 1:**
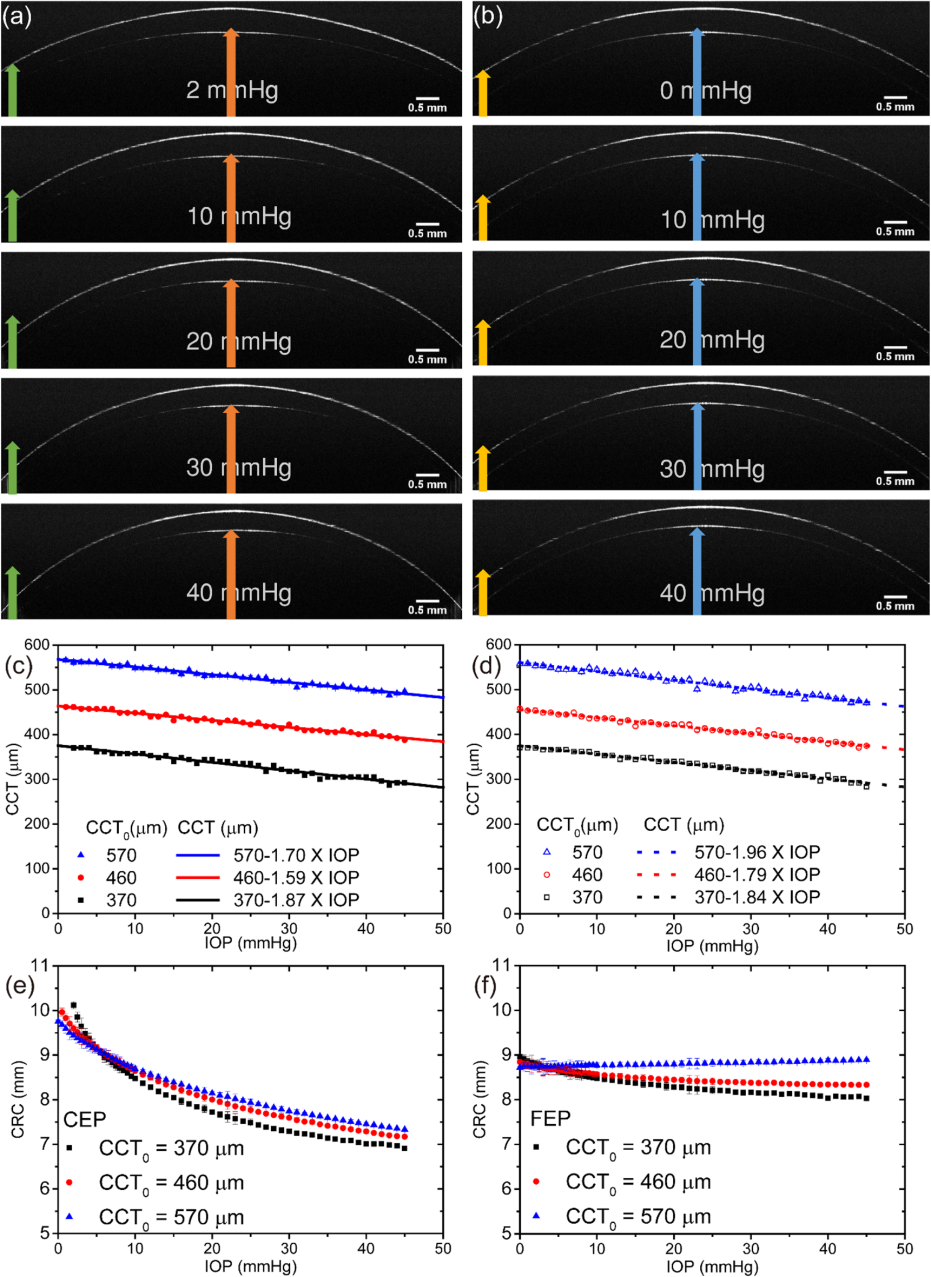
Optical coherence tomography images of cornea eyeball phantom (**a**) and full eyeball phantom (**b**) during changes in applied pressure. The CCT_0_ of both phantoms is 370 μm. The scale bar is 0.5 mm. Arrows of the same color are the same size. (**c**) and (**d**) show the changes in the central corneal thickness of CEP and FEP, respectively. (**e**) and (**f**) show the corneal radius curvature for CEP and FEP, respectively, with changes in the applied pressure.

OCT images as a function of IOP are shown in Fig. 1(a,b) for the CEP and FEP, respectively. The phantoms were designed to have a CCT at zero IOP (CCT_0_) of about 350 μm. For both eye phantoms, CCT decreased considerably with increasing IOP. The corneal radius of curvature (CRC) for the CEP decreased with increasing IOP, whereas an increase in the IOP of the FEP resulted in a less prominent decrease in the CRC. We took OCT images of the cornea with IOP changes at every 1 mmHg interval as the IOP increased from 0 to 45 mmHg, and we estimated both the CCT and the CRC (see Supplementary Fig. S1(a,b)). The results for the CEP and FEP are shown in Fig. 1(c,d), respectively. The CCT decreases linearly as the internal pressure increases. The slope of the plot was almost independent of the type of eye phantom (see Table 1). The average slope for the CEP was approximately $-1.72\pm 0.14$ μm/mmHg while the slope for the FEP was approximately $-1.86\pm 0.09$ μm/mmHg. Because the slopes for the two types of eye phantoms were within one standard deviation of each other, it is reasonable to conclude that the dependence of the CCT on the IOP may be virtually the same between the two models. In addition, CCT_0_, determined by linear regression, was fairly consistent with the initial designs for the CCT.

**Table 1:** A comparison of the overall changes in central corneal thickness (CCT) and cornea radius curvature (CRC) and the difference in non-contact tonometer (NCT) readings according to the initial CCT and the types of eye phantoms.

${\mathrm{CCT}}_{0}$(μm)	Slope of the plot of CCT versus IOP (μm/mmHg)		Total change in CRC (mm)	
	CEP	FEP	CEP	FEP
370	− 1.87	− 1.84	− 3.21	− 0.93
460	− 1.59	− 1.79	− 2.79	− 0.51
570	− 1.7	− 1.96	− 2.42	0.17
${\mathbf{C}\mathbf{C}\mathbf{T}}_{0}$**(μm)**	**Slope and intercept of the plot of NCT readings versus IOP**			
	**CEP**		**FEP**	
	**Slope**	**Intercept (mmHg)**	**Slope**	**Intercept (mmHg)**
370	0.744	− 0.598	0.552	2.04
460	0.887	4.06	0.577	5.98
570	0.950	10.4	0.611	12.1

*IOP* intraocular pressure, *CEP* cornea eye phantom, *FEP* full eyeball phantom, ${CCT}_{0}$ CCT under zero IOP.

**Figure 2:**
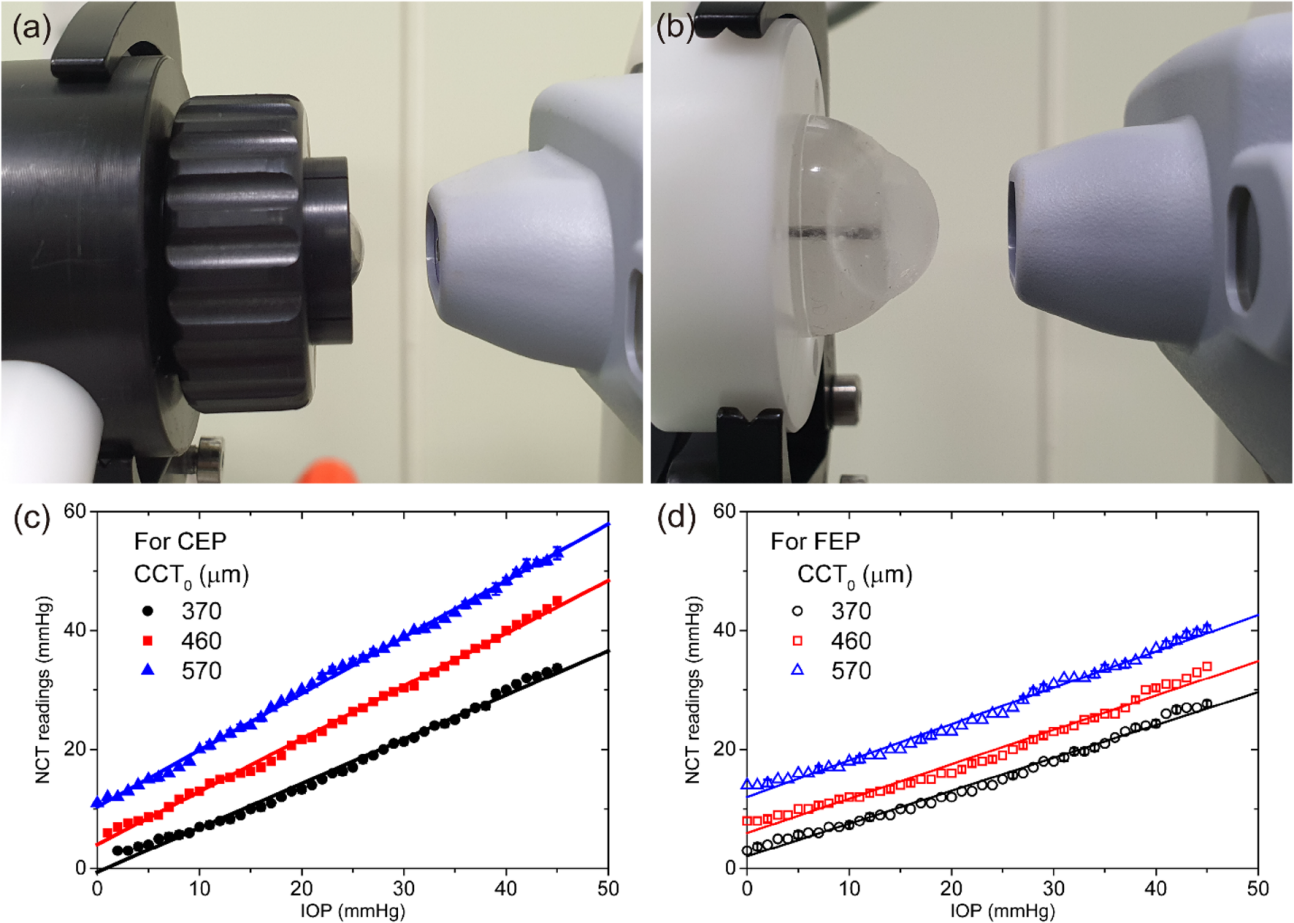
The experimental setup for measuring intraocular pressure with non-contact tonometer for the cornea eyeball phantom (**a**) and the full eyeball phantom (**b**). The relationships between the NCT readings and the IOP for the cornea and full eyeball phantoms are shown in (**c**) and (**d**), respectively.

Meanwhile, as shown in Fig. 1(e,f), the relation between the CRC and the IOP increment differed considerably between the CEP and the FEP. For the CEP with CCT_0_ values of 370 μm, 460 μm, and 570 μm, CRC decreased as the IOP increased. The degree of changes in the CRC decreased as CCT_0_ increased. The CRC of the FEP designs with CCT_0_ values of 370 μm and 460 μm exhibited a negative correlation with IOP. However, for the FEP with a CCT_0_ of 570 μm, the CRC increased with increasing IOP. It should also be noted that the dependence of the CRC on the internal pressure for the FEP was much less than that for the CEP (*p* < 0.05). The overall changes in CRC for both model eyes as the IOP increased from 0 to 45 mmHg are shown in Table 1. We further investigated the dependence of the tonometer readings on IOP by using NCT. Figure 2(a,b) show the photographs taken during the NCT readings for CEP and FEP, respectively. As shown in Fig. 2c, the NCT readings for the CEP were positively correlated with IOP. The NCT readings were linearly dependent on the IOP. The slopes and intercepts estimated from linear regression formula for the eye phantoms with CCT_0_ values of 370 μm, 460 μm, and 570 μm are shown in Table 1. The slope should be one if the current NCT readings are ideal for the estimation of the IOP inside the eye phantoms. Both the slope and intercept of the plot increased with increasing CCT_0_. This may reflect the relative modulus changes accompanying the CCT_0_ changes. Thicker eye phantoms showed a higher NCT readings. Plots of the NCT readings versus IOP for FEPs with three different CCT_0_ values are shown in Fig. 2d. The slopes and intercepts of the plots, shown in Table 1, increased as CCT_0_ increased, similar to what was observed in CEP. For all CCT_0_ values, the slope of the FEP plot was less steep than that of the CEP plot.

**Figure 3:**
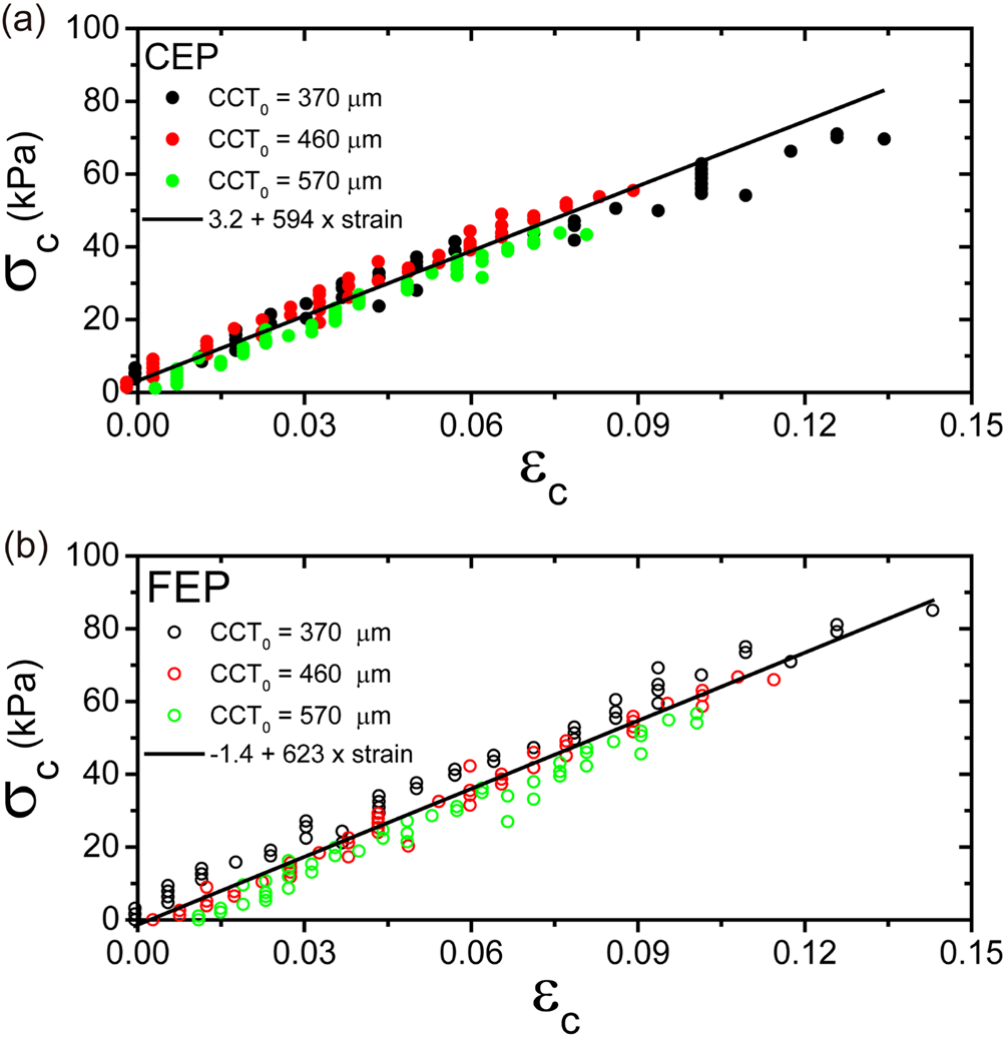
Circumferential stress (σ_c_)–circumferential strain (ε_c_) curves for the cornea eyeball phantom (**a**) and the full eyeball phantom (**b**) derived from the data shown in Fig. 1(**c**–**f**). The solid black lines are the results of the linear regression conducted on all of the data.

The circumferential (or tangential) strain accounts for the in-plane strain of the corneal surface of the phantom because the stress in the thin wall of the pressurized eyeball phantoms is one of the in- plane stresses. The circumferential stress and circumferential strain could be estimated from the data shown in Fig. 1(c,d,e,f). The relations observed from the CEP and FEP are shown in Fig. 3(a,b), respectively. In both eye models, we were unable to detect any apparent dependence of the slope on the CCT_0_. All of the data were quite linear when the circumferential strain was small. From the circumferential stress– circumferential strain curves, the slopes for the CEP (Fig. 3(a)) and FEP (Fig. 3(b)) were estimated to be 594 ± 16 kPa and 623 ± 12 kPa, respectively. The Young’s modulus (E) representing the linear elasticity of PDMS, which is the material used to fabricate the eye phantoms, was determined to be 380 kPa. If both the slope of the curves and the Young’s modulus of PDMS were accurate, the range of Poisson’s ratio were found to be 0.36 ± 0.02 and 0.39 ± 0.02 for the CEP and FEP, respectively.

**Figure 4:**
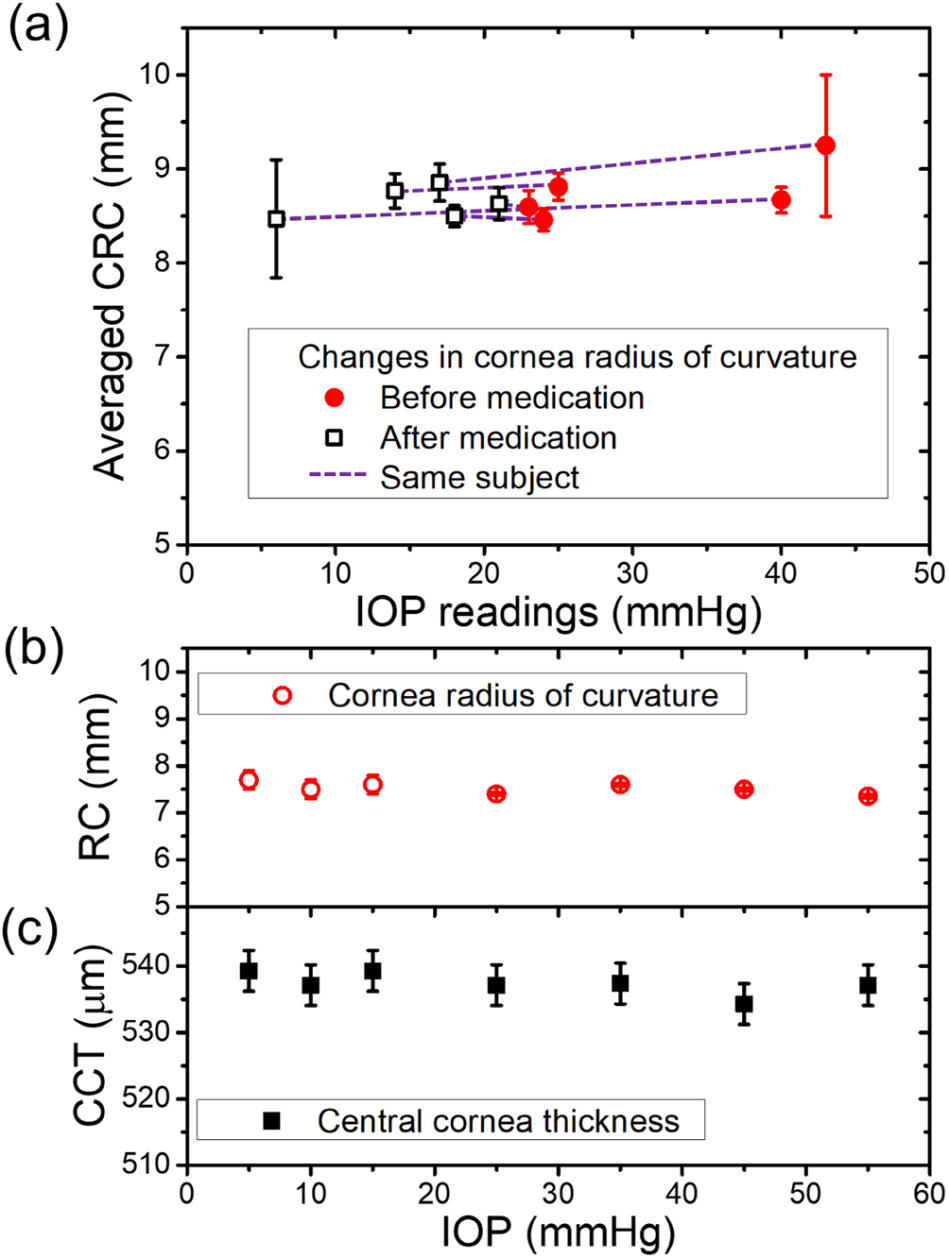
The deformation of human subjects governed by intraocular pressure changes. The averaged corneal radius curvature of human eye before (closed red circle) and after (open black square) medications is displayed in (**a**). The data pair connected by dashed line is measured from the subject of the same patient. The IOP were measured by NCT 4 h after medications. The dependence of the cornea radius of curvature and the central corneal thickness on IOP observed from the cannulated human subject are shown in (**b**) and (**c**), respectively. The measurement for each IOP change is completed within 1 min.

The deformation of human eyes induced by IOP changes was investigated by both non-invasive and invasive ways. Figure 4(a) shows the CRC averaged over all scanned corneal surface from human eye before medication (closed red circle) and 4 h after treatment (open black square) to recover to normal range IOP. The data pair connected by dashed line is measured from the subject of the same patient. Even if the number of studied human subjects is rather limited, it should be noted that the CRC has a positive correlation with IOP. Figure 4(b,c) show the dependence of the CRC and CCT on IOP observed from the cannulated human subject, respectively. It is reasonable to suppose that within experimental error limit, the CRC and CCT of human subjects treated with *pars plana* vitrectomy is hardly affected by IOP change. It should be noted that the values of CRC and CCT shown in Fig. 4(b,c) are determined from OCT cornea images within 1 min after IOP change while the results shown in Fig. 4(a) observed from the human subjects more than 4 h after medication treatment. Because of limited knowledge about detailed restoration dynamics for the corneal structural characteristics for human eye caused by IOP change, it is difficult to make any conclusion to determine which of the experimental results of noninvasive medication shown in Fig. 4(a) and invasive ones shown in Fig. 4(b,c) is correct. At any rate, we are able to reasonably suggest that the observations from the human subjects under in vivo conditions are much more similar to the structural deformation of FEP shown in Fig. 1(b,d,f).

## Discussion

Deluthault et al.^7^ developed a silicone eye phantom similar in shape to a contact lens, and used it to test a glaucoma diagnosis device. The Young’s modulus of the phantom cornea, a measure of its linear elasticity, was approximately 2.5 MPa, which differed from the physiological Young’s modulus of the cornea^15^. The CCT and CRC were approximately 210 µm and 8.2 mm, respectively, which were also inconsistent with those of real eyes. The CCT and the CRC of normal human corneas are in the range of 400∼600 μm^16–18^ and 7 ∼ 8 mm^16,18^, respectively. Hsu et al.^8^ developed an eye phantom for long-term IOP monitoring to test a lens-type sensor system. This eye phantom mimicked the pressure changes by using a micropump. Khan et al.^19^ developed an eye phantom with viscoelastic material for corneal elastography measurements. The CRC of this corneal phantom was 8.4 mm. Ko et al.^20^ developed a silicone eye phantom to characterize the corneal tangent modulus. The CCT and CRC of this eye phantom were 1100 µm and 8 mm, respectively. These phantoms all belong to the modular type. The cornea of these models is made of an elastic polymer to mimic the flexible tissues of a human cornea, while the other parts are fabricated from solid materials. The artificial cornea is usually fixed to rigid parts. These “fixed-cornea” eye phantoms have the advantage of being easy to fabricate and replace with materials of different structural and mechanical properties. However, there is no evidence that these CEPs can accurately mimic the structural deformation of the human eye. If the IOP increases or an external force is applied to the human eyeball, deformation occurs throughout the eyeball to maintain equilibrium. In contrast, in the case of a fixed-cornea eye phantom, any deformation occurs only in the cornea because the other parts of the eye phantom are rigid. For this reason, the deformation of a CEP in the presence of IOP changes and/or an external force would be quite different from that of a human eye. Furthermore, as the tension of the cornea is measured from the outside, the estimation of IOP could be affected.

**Figure 5:**
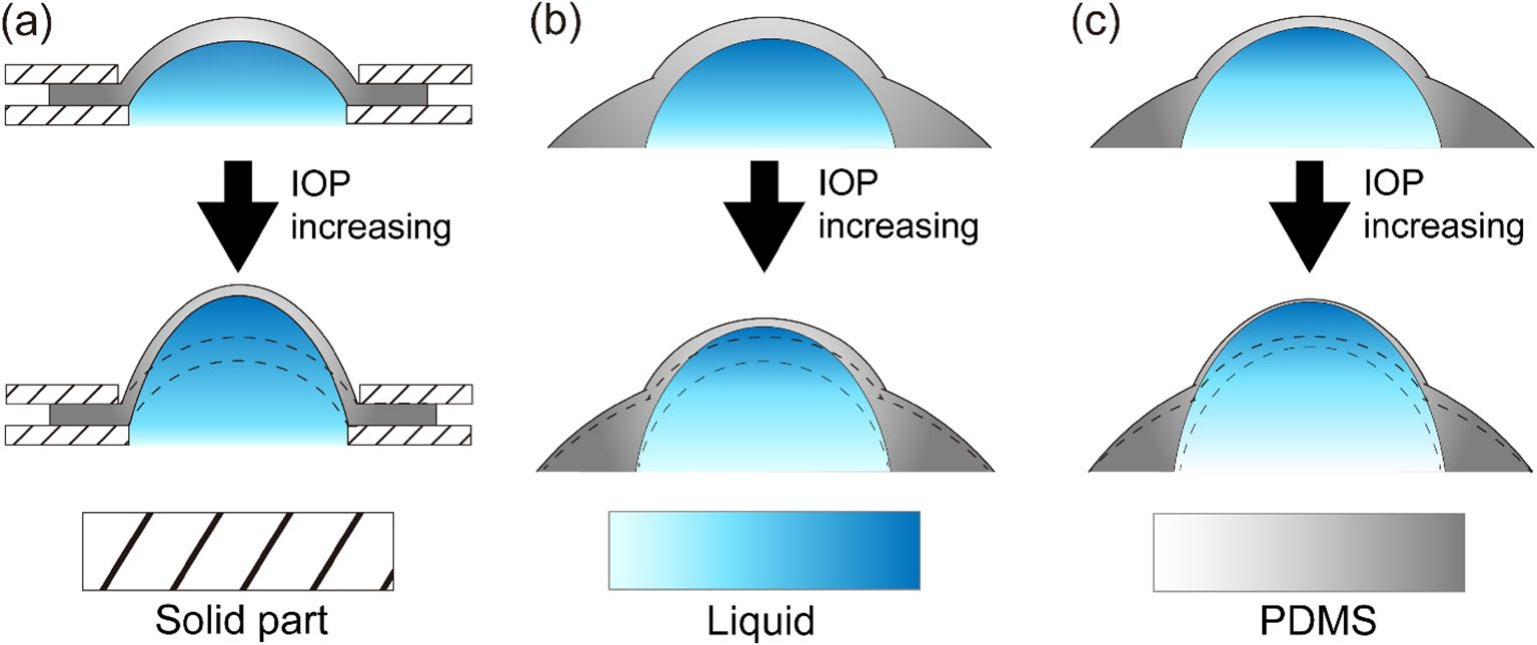
Illustration describing the structural deformation caused by increasing intraocular pressure for the cornea eyeball phantom (**a**) and the full eyeball phantom with thick (**b**) and thin (**c**) the central cornea thickness under zero IOP.

In the present study, we investigated the structural deformation of the two different types of eye phantoms at varying IOPs. NCT readings were also performed with an air-puff tonometer, which is the device most commonly used in clinical practice for glaucoma screening to obtain information on the changes in the tension of the cornea from the outside. To analyze the structural changes in the eye phantoms quantitatively, we estimated the CCT and CRC from the OCT images (see Supplementary Fig. S1). We found that the relation of the CCT and CRC to the IOP differed significantly between the FEP and CEP models. While the CCT changes were very similar between the two models, the relation between the CRC and the IOP was dependent on the type of eye phantom. For the CEP, the CRC drastically decreased as internal pressure increased (see Fig. 1 and Table 1). For the FEP, however, we found that the changes in CRC as a function of changing IOP was dependent on CCT_0_. When CCT_0_ was less than 460 μm, the CRC decreased as IOP increased. However, when CCT_0_ was 570 μm, the CRC increased as IOP increased. When the IOP increases, deformation of the cornea should be mainly governed by its stiffness, which is determined by its material properties, structure, and boundary properties. At the boundary between the flexible and rigid parts of the CEP, the cornea extended significantly as the applied pressure increased under the constraint condition. This causes the axial length of the eye phantom to increase and there was an eventual decrease in the CRC accompanied by a decrease in CCT (Fig. 5(a)). In addition, CRC was dependent on CCT_0_. The cornea axially extended with decreasing CCT_0_. Among all of CEPs, changes in the CRC of the CEP with a CCT_0_ of 370 μm were most prominent. Thus, in the case of a thin cornea, IOP induces remarkable structural changes near the corneal apex. Meanwhile, the entire area of the FEP could be deformed by IOP changes even if the stiffness of the eyeball part except the cornea was relatively high. While the decrease in the CCT for the FEP was comparable to that of the CEP upon applying pressure, the change in CRC in the FEP was less prominent than that in the CEP. When the CCT_0_ was 570 μm, the CRC was either constant or increased because most of the applied pressure contributed to compression of the CCT (Fig. 5(b)). Meanwhile, if the initial CCT was less than 460 μm, the CRC of the FEP slightly decreased when the internal pressure increased (Fig. 5(c)). Based on data from 1390 people, Shimmyo et al.^16^ reported a significant correlation between CCT and keratometric power: thicker eyes had a flatter CRC and thinner eyes had a steeper CRC. In addition, by using enucleated human eyeballs, Hjortdal et al.^21^ reported an apparent increase in CRC as IOP increased. The results on in-vivo human subjects from this work also apparently reveal that the dependence of the CRC of the human subjects on IOP is similar to that observed from FEP. (see Fig. 4) Although we have no information on the CCT_0_ of human subjects of the abovementioned studies, this led us to reasonably suppose that the FEP with a CCT_0_ of 570 μm is the most suitable model to describe the deformation of the human eye during an increase in IOP. When the IOP increases, the corneal surface expand and the CCT decreases due to the force caused by the IOP.

Assuming that we set the region of interest near the corneal apex, the system can be considered to be part of a thin-walled spherical shell (see Supplementary Appendix). As the CCT is much lower than the CRC, the circumferential stress (σ_c_) can be expressed as follows^22^:

1 $$\sigma_{c} = \frac{IOP \times CRC}{{2 \times CCT}}$$

Assuming that the volume of a finite element at the corneal apex is constant, when the strain is quite small, the circumferential strain (${{\varvec{\varepsilon}}}_{{\varvec{c}}}$) can be expressed in terms of CCT as follows:

2 $$\varepsilon_{c} = \left( {\frac{{CCT_{0} }}{CCT}} \right)^{1/2} - 1$$

Furthermore, Hooke’s law states that

3 $$\frac{E}{{\left( {1 - \nu } \right)}} = \frac{{\sigma_{c} }}{{\varepsilon_{c} }}$$

where, E and ν are the Young’s modulus and Poisson’s ratio, respectively.

Even if an information about the Poisson's ratio of the PDMS used in the construction of eye phantoms remains rather limited, the values might be close to the recently reported values (range, 0.40–0.50)^23^. In the case of the human cornea, the mean Poisson’s ratio is 0.42^24^. For isotropic and homogeneous materials, the relationship between the Young's modulus (E) and the shear modulus (G) is E = 2G(1 + ν). Therefore, the shear modulus can be estimated as approximately 93 kPa and 91 kPa for the CEP and the FEP, respectively. The values determined from our eye phantoms were close to the shear modulus of the human cornea, G = 72 ± 13.7 kPa^23^. These observations led us to propose that our eye phantoms could be utilized to investigate deformation of the eye caused by IOP changes or to measure the tension from outside the eyeball. Indeed, a recent study on the biomechanics of an in vivo human cornea showed that the corneal shear modulus was independent of both CCT and IOP^12^. This is consistent with the observations shown in Fig. 3. The slope of circumferential stress (σ_c_) – circumferential strain (ε_c_) plot is independent of both CCT of eye phantom and IOP.

We found that the tonometer readings of eye phantoms tended to increase as the initial CCT changed. Even if an air-puff tonometer overestimates or underestimates the true IOP^25^, the observation from the current work is consistent with previous reports on the positive correlation between the tension measured from outside the eyeball and the CCT^16^. Meanwhile, the increment in the NCT readings due to the increase in IOP from 5 to 45 mmHg was approximately 1.5 times greater in the CEP than in the FEP (Table 1). These discrepancies might be due to the difference in their structural deformation as the applied pressure increased. The CRC of the CEP decreased significantly as the IOP increased. This configuration made the cornea more difficult to flatten, and eventually, it exhibited higher NCT readings because the readings are derived from the force needed to flatten the cornea. The changes in CRC with increasing IOP were lower in the FEP than in the CEP. Therefore, the ratio of NCT readings to IOP changes should be lower in the FEP than in the CEP.

We evaluated whether the FEP or the CEP better mimics the human eyeball and is more suitable for understanding the relationship between the tension measured from outside and the structural deformation of the eyeball induced by an increment of IOP. It is challenging to measure both CCT_0_ and CRC, which are correlated with structural deformation, in human subjects at an IOP of 0 mmHg. In addition to the observations in in-vivo human subjects from this work, enucleated human eyeballs and large population data studies^17,21^, it is crucial to refer to theoretical studies on deformation of the cornea upon the application of IOP. A theoretical simulation study^24^ on the two types of eye models revealed an apparent increase in CRC with increasing in IOP in an FEP with a relatively thick cornea. However, CRC decreased with increasing in IOP in the CEP. These considerations enable us to suggest confidently that the FEP more closely recapitulated the structural deformation of the human eye in response to IOP change. Comparison with systematic in-vivo studies on the structural deformation caused by IOP alteration in human subjects in large scale should confirm whether the results from the current work imply the possibility that an FEP is suitable for performance testing of tonometers.

## Conclusion

We have conducted a comparative study on IOP induced cornea deformation on two different eye phantoms of FEP and CEP as well as in vivo human subjects. We developed our own method to fabricate the FEP. Subsequently, we investigated the structural changes accompanied by IOP alteration to get a quantitative information on the CCT, CRC, and NCT readings. By referring to the results from in vivo studies as well as the previous studies on structural deformation using both in vitro experiments of the human eye and theoretical considerations, we suggest that our FEP mimics the human eye more accurately than a CEP. From the NCT measurements, we also found that the NCT readings from the CEP were overestimated, while those from the FEP were not. For these reasons, we supposed that the FEP could be suitable for the estimation of true IOP and allow performance testing of tonometers for medical checkups and other clinical uses once further studies confirm that the FEP recapitulates the results of in-vivo studies on human subjects.

## Methods

### Fabrication of eye phantoms

**Figure 6:**
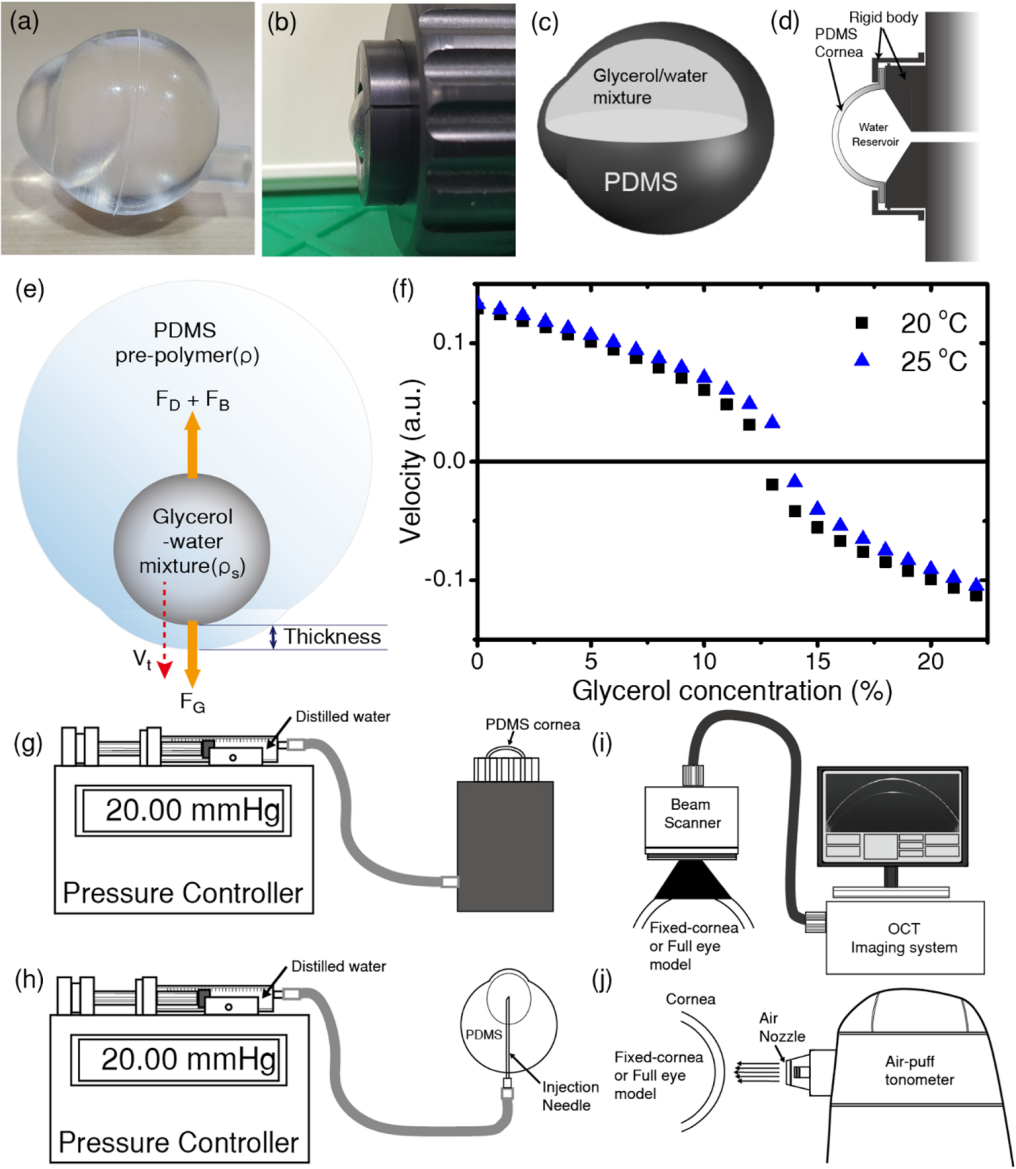
Photographs of the full eyeball phantom (**a**) and cornea eyeball phantom (**b**) and their cross-sectional views of the two eye phantoms are shown in (**c**) and (**d**), respectivel. Diagram of a glycerol-water mixture in polydimethylsiloxane (PDMS) prepolymer (**e**). In this case, the density of the glycerol-water mixture (${\rho }_{s}$) is higher than that of PDMS prepolymer ($\uprho$). The relationship between the glycerol concentration of the glycerol-water mixture and the velocity of the glycerol-water mixture drop in the PDMS prepolymer when the ambient temperature is 20 °C and 25 °C (**f**). When the glycerol concentration is near 13%, there is a singular time-point at which the drop of glycerol-water mixture changes its direction of movement. Syringe pump-based pressure control system for the cornea eyeball phantom (**g**) and full eyeball phantom (**h**). The schematic diagrams for measuring the structural deformation with optical coherence tomography and non-contact tonometer readings of the cornea are shown in (**i**) and (**j**), respectively.

We developed two different types of eye phantoms. Figure 6(a,b) show photographs of the FEP and CEP, respectively. The detailed design of the phantoms shown in Table 2 is based on the characteristics of the human eyeball including its dimensional size^26,27^, CCT^16–18^, corneal diameter^18,28^, and CRC^16,18^. The manufactured FEP and its internal structure are shown in Fig. 6(a,c), respectively. The FEP has a spherical shell structure made of PDMS filled with a glycerol-water mixture. The cornea of the CEP was fabricated by curing a mixture of the base elastomer and a curing agent inside an appropriate mold made of polyethylene terephthalate (PET). The corneal phantoms were fixed to the rigid components with a water reservoir (Fig. 6(d)). Both the initial CCT and CRC of the phantom were designed by adjusting the geometry of the mold.

**Table 2:** The features of the two types of eye phantoms.

Feature	Full eyeball phantom(FEP)	Cornea eyeball phantom(CEP)
Central corneal thickness (μm)	370–570	370–570
Young’s modulus (kPa)	380	380 (only cornea)
Corneal radius of curvature (mm)	8.5	8.5
Cornea diameter (mm)	11	11
Materials of the eye phantoms	Polydimethylsiloxane	Cornea: Polydimethylsiloxane Other parts: rigid plastic

The CCT of the FEP was controlled by varying the density of the glycerol-water mixture. The mold was made of PET. A liquid PDMS mixture was poured into the mold. Afterward, the aqueous glycerol solution was injected into the liquid PDMS mixture. Because the PDMS mixture is immiscible with the aqueous mixture, the movement of the droplet of the mixture is governed by the following three forces (see Fig. 6(e)).

4 $${\text{Gravitational force:}}\;{\text{F}}_{{\text{G}}} { = }\frac{{\uppi }}{{6}}{\text{d}}^{{3}} {\uprho }_{{\text{s}}} {\text{g}}$$

5 $${\text{Buoyancy force:}}\;{\text{F}}_{{\text{B}}} { = }\frac{{\uppi }}{{6}}{\text{d}}^{{3}} {\rho g}$$

6 $${\text{Drag force:}}\;{\text{F}}_{{\text{D}}} {\text{ = c}}_{{\text{d}}} \frac{{1}}{{2}}{\text{V}}^{{2}} {\uprho }$$

where, ${\varvec{d}}$ is the diameter of the aqueous glycerol solution, *g* is the gravitational acceleration, and ${\varvec{\rho}}$ and ${{\varvec{\rho}}}_{{\varvec{s}}}$ are the densities of the PDMS prepolymer and aqueous glycerol solution, respectively. A is the projected area of the droplet of the glycerol-water mixture, ${{\varvec{c}}}_{{\varvec{d}}}$ is the drag coefficient, and V is the velocity relative to the object. In a steady state, the sum of the forces should be zero.

7 $${\text{F}}_{{\text{G}}} {\text{ = F}}_{{\text{B}}} {\text{ + F}}_{{\text{D}}}$$

The droplet of the glycerol-water mixture reaches the terminal velocity (${{\varvec{V}}}_{{\varvec{t}}}$), which can be expressed as follows:

8 $$V_{t} = - \sqrt {\frac{4gd}{{3C_{d} }}\left( {\frac{{\rho_{s} - \rho }}{\rho }} \right)} \cdots \rm when\;\rho_{s} > \rho$$

9 $$V_{t} = \sqrt {\frac{4gd}{{3C_{d} }}\left( {\frac{{\rho - \rho_{s} }}{\rho }} \right)} \cdots \rm when\;\rho_{s} < \rho$$

Both the speed and the direction of movement of the drop can be controlled by varying the density of the glycerol-water mixture and its injected volume. The density of the glycerol-water mixture was adjusted by changing its composition. The changes in the terminal velocity (Eq. (8) and (9)) can be controlled by adjusting the concentration of glycerol in the aqueous mixture as shown in Fig. 6(f). When the concentration of glycerol was 13%, the terminal velocity of the drop was zero. A drop, whose density is greater than that of the PDMS mixture, will tend to sink. If the drop is less dense than the liquid PDMS mixture, the buoyancy force can keep the drop afloat. Because the density and drag coefficient of the liquid of the PDMS prepolymer depend on the degree of cross-linking reaction, the terminal velocity of the drop also changes. Supposing that the curing condition is well designed and controlled for all trials to fabricate the phantom, a vertically moving drop will come to rest at a specific point, whose final position depends only on the nature of the drop. This means that the thickness between the bottom of the mold and the underside of the drop can be adjusted by varying both the density and volume of the drop of the glycerol-water mixture. Based on this principle, we made a three-dimensional FEP with adjustable corneal thickness. The CCT of the FEP could be successfully controlled in the range from 200 to 550 μm with changing the concentration of glycerol in glycerol-water mixture from 22 to 10%.

The eye phantoms used in this work were designed to have CCT values of 350 μm, 450 μm, and 550 μm, which are close to the average CCT of the human eyeball. After fabrication, we found that the CCT values with no applied force were 370 μm, 460 μm, and 570 μm. In addition, the CRC of the eye phantoms was designed to be 8.5 mm. The Young’s modulus of the PDMS phantom materials was adjusted to approximately 380 kPa by controlling the composition of the liquid PDMS mixture (Sylgard 184, Dow Corning, the weight ratio of the silicone elastomer to the curing agent was 22:1)^29^. The Young’s modulus, representing the linear elasticity, was measured by a tensile method^30^. PDMS samples for tensile stress testing were prepared according to the ASTM D 412 standard. Dog-bone shaped samples with cross sections of 7 × 3 mm^2^ were used to measure the Young’s modulus of the PDMS samples. All the measurements were carried out within 3 days after the production of the phantoms.

### Experimental setup for controlling IOP

A syringe pump-based pressure control system (C&V Tech, Korea) was used to control the internal pressure of the eye phantom. The CEP was connected to the pressure control system through a flexible tube (Fig. 6(g)). The IOP of the FEP was adjusted by directly injecting the water using a 23-gauge needle (Fig. 6(h)). Before performing the experiment, the aqueous glycerol solution in the FEP is replaced with pure water. The height at the top of the cornea was kept the same as that of the syringe to maintain the same pressure. The IOP inside the eye phantom was measured using a calibrated pressure sensor (PSH-B0200, Sensys), whose height was kept the same as that of the apex of the cornea. The measurement of internal pressure in the eye phantoms was repeated three times as the pressure was raised and lowered to determine whether there was any possible hysteresis. As shown in Supplementary Fig. S2(a), the intercept and the slope obtained from the plot of the measured pressure versus the set value are approximately 0.31 ± 0.11 mmHg and 1.000 ± 0.002 mmHg, respectively. We captured OCT images of the cornea (Fig. 6(i)) while varying the internal pressure near zero because it is crucial to find the exact zero point of IOP to accurately determine the corneal deformation with changing internal pressure. As shown in Supplementary Fig. S2(b), the cornea apparently weaved under negative pressure and return to a circular surface shapes if the IOP increased to 0 mmHg. These observations mean that the current experimental configuration is suitable for investigating the deformation of the eye phantom with high accuracy.

### Measurement

The OCT images (Fig. 6(i)) and NCT readings (Fig. 6(j)) for the CEP were observed by fixing the rigid outer part of the water reservoir. In the case of the FEP, the needle, which is used to control the IOP inside the model eyeball, is the only fixed part. A custom-built hemispherical cup-shaped holder maintained the orientation of the phantoms during measurement. By using this configuration, we were able to minimize the constraints imposed by the fixation of FEP phantoms except the positioning of the injection needle. To examine the corneal deformation, tomographic images were obtained with a spectral domain optical coherence tomography (SD-OCT) system (Telesto, Thorlabs, USA) as shown in Fig. 6(i). The eye phantoms were positioned upright (cornea side up). The center wavelength and wavelength range were 1300 nm and 100 nm, respectively. The probe beam was scanned with a Galvano scanner at a frame rate of approximately 18 f/s. The range of B-scans, consisting of 4096 A-scans, was 10 mm. A refractive index of 1.00 was set for all the measurements. The resolution and the range in the axial direction were approximately 4.9 μm and 2.528 mm, respectively. Both CCT and CRC were obtained by analyzing the OCT images with data-processing code written in Python. The anterior and posterior surfaces of the cornea from the OCT image were extracted by using an edge detection algorithm. The CCT was estimated by dividing the distance between the two surfaces at the apex of the cornea by 1.4, the refractive index of PDMS (Supplementary Fig. S1(a)). CRC was estimated by fitting the anterior surfaces with a circular function (Supplementary Fig. S1(b)). An air-puff tonometer (CT-80, Topcon) was used to collect NCT readings from the eye phantom as the IOP varied from 0 to 45 mmHg. As shown in Fig. 6(j), the eye phantoms were oriented parallel to the direction of the air jet, which was kept perpendicular to the corneal surface. All of the measurements for NCT readings and OCT images were repeated three times as the pressure was raised and lowered to determine whether there was any possible hysteresis. There were no observable changes in these measurements depending on the direction of the pressure changes.

### In vivo study design for human subjects

Volunteer patients seeking treatment at the Wonkwang University School of Medicine were recruited. This study followed a protocol approved by Institutional Review Board (IRB) and in accordance with the principles of the Declaration of Helsinki. Written informed consent was obtained from all subjects and/or their laga guardians, The age of the subjects ranged from 53 to 79 years. For in vivo studies on human subjects, we performed both noninvasive and invasive ways. Glaucoma is characterized by high IOP and should be decreased to normal with medication of topical and/or systematic drug prescription without any invasive maneuver to the eye. Individuals with an IOP beyond 60 mmHg before medicinal treatment were excluded from the study due to corneal edema and haziness. CRC as well as IOP of three patients with secondary glaucoma and two patients with primary open angle glaucoma were investigated before and after medication by using a refractive power/corneal analyzer (OPD-Scan III, NIDEK, Japan) and a pneumatic tonometer (KT-980, KOWA, Japan), respectively. CRC and IOP were measured at least 4 h after drug treatment. Invasive studies on the human subject was carried out during *pars plana* vitrectomy operation after insertion of infusion cannula. The IOP inside eye was controlled through infusion cannula, which is inserted into the sclera of eyeball. After changing the intraocular pressure to 5 mmHg, 10 mmHg, 15 mmHg, 25 mmHg, 35 mmHg, 45 mmHg and 55 mmHg (Stellaris PC, Bausch & Lomb, USA), OCT images of the cornea were taken and used to determine both CRC and CCT.

## Supplementary Information

Supplementary Information.

## Data Availability

All data generated or analyzed during this study are included in this published article and its supplementary Information files.
