# Supplementary material for: Development of eye phantom for mimicking the deformation of the human cornea accompanied by intraocular pressure alterations
Source: Sci Rep. 2022 Nov 30;12:20670. doi: 10.1038/s41598-022-24948-2 (PMC9712605; doi:10.1038/s41598-022-24948-2)
Supplement: Supplementary file 1 — Supplementary Information. [file 41598_2022_24948_MOESM1_ESM.docx]

**Supplementary Figure S1**


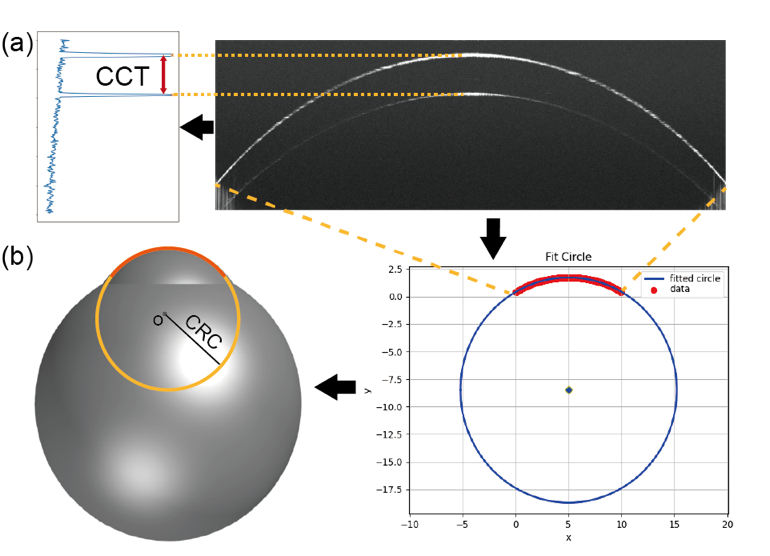


# Figure S1. The anterior and posterior surfaces of the cornea were extracted by using the edge detection algorithm. (a) The CCT was estimated by dividing the distance between the two surfaces at the apex of the cornea with PDMS refractive index of 1.4. (b) CRC was estimated by fitting the anterior surfaces with a circular function.

# Supplementary Figure S2


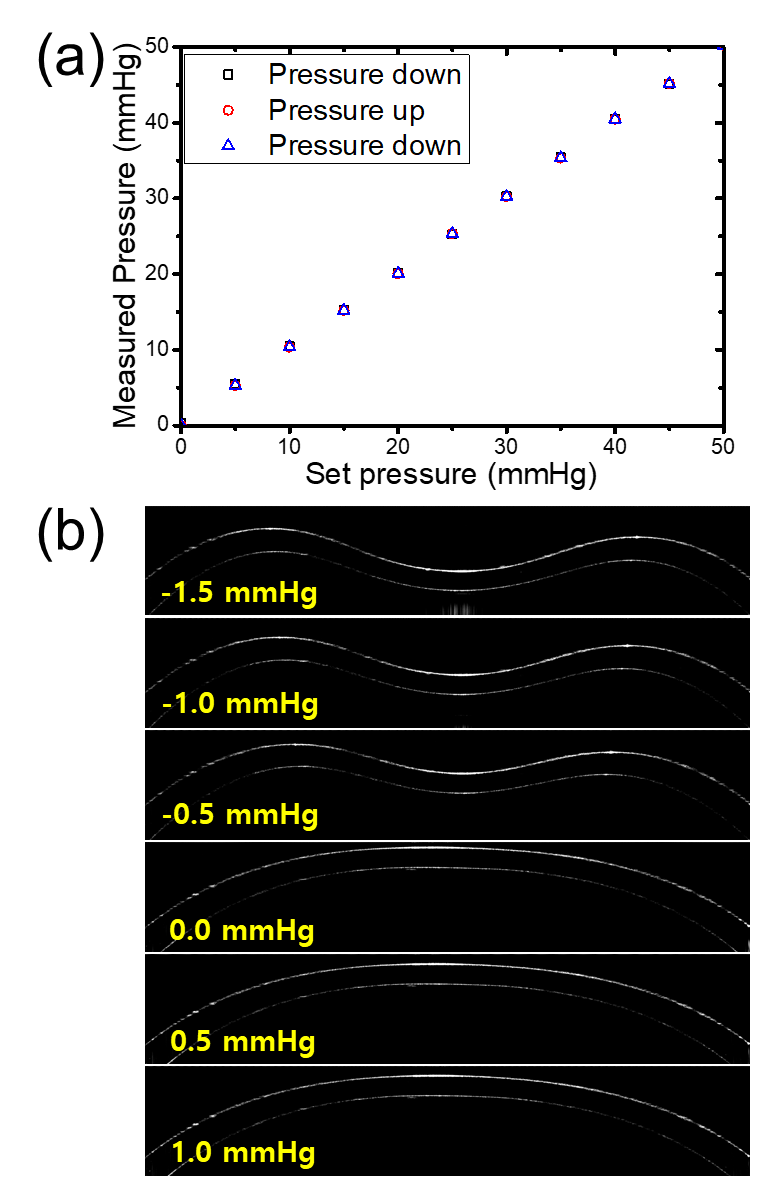


**Figure S2.** The plot of the measured pressure inside eye phantom versus the set pressure of the pressure controller (a) and the OCT images of the cornea (b) with changing the internal pressure from -1.5 mmHg to 1.0 mmHg with a step of 0.5 mmHg. There is no any apparent difference between the pressure measurements with changing the direction of the IOP change. The contour of the cornea of the eye phantom apparently weaved if the IOP is negative pressure. However, the cornea changes to have circular-shaped surface if the IOP increases to 0 mmHg.

# Supplementary Appendix

# 1. Mechanical model for the cornea deformation

- We can consider the corneal part of the phantom (Fig. S3(a)) as a thin spherical shell, and the force balance equation (Fig. S3 (b) and (c)) can be written with the corneal radius of curvature (CRC) and central corneal thickness (CCT) as follows^1, 2^ :

$IOP\times\pi\times{CRC}^{2}=\sigma_{C}\times2\pi\times CRC\times CCT$ (S1)

in which IOP is an intraocular pressure. From equation (S1) the circumferential stress can be described with the measurable variables of the corneal characteristics and applied pressure of IOP.

$\sigma_{c}=\frac{IOP\times CRC}{2\times CCT}$ (S2)

#
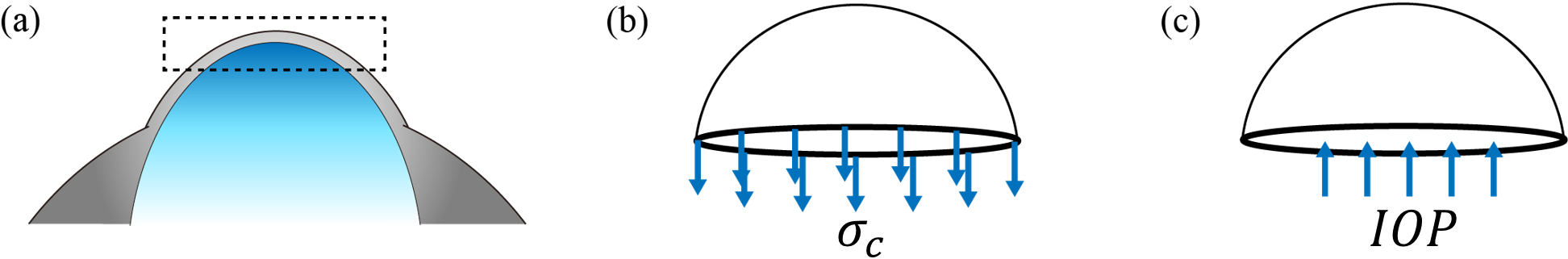


**Figure S3.** (a) Cross-sectional diagram of the cornea part of the eyeball phantom. The free body diagram of the cornea consists of two different forces: (b) circumferential stress (σ_c_) and (c) intraocular pressure (IOP). These forces should be equilibrated to maintain the structure of the eyeball by changing the applied forces.

The thin-walled pressure vessel model has been generally regarded as a reasonable approximation for the analysis of the mechanical response of the cornea accompanied by changes in IOP.^3^ In this model, the cornea should be considered as an elastic and isotropic spherical shell. The measured variable with changing the IOP is the apical increments at the center of the cornea. The values were used to calculate the circumferential strain ε_c_,

$\varepsilon_{c}=\frac{\delta r}{r}$ (S3)

where $r$ is the mid-radius of the thin-shell. $\delta r$ is the increment of the radius accompanied by increasing the internal pressure. The geometrical changes of thin spherical shell under 0 mmHg (Fig. S4(a)) and IOP mmHg is describe in Fig. S4(a) and S4(b), respectively. In fact, the shape of the cornea of both human subjects and the phantoms is not purely spherical. In addition, the center of the corneal curvature should be moved when the eyeball expands radially with an increase in IOP. Furthermore, the central cornea is subjected to out-of-plane strain changes. From the current experiments with some of the full eyeball phantom and all of human subjects, we observed that the CRC decreased with increasing IOP accompanied with a rise in the corneal apex. This means that the aforementioned model could not be applied to the analysis of the mechanical properties of our eye phantoms.


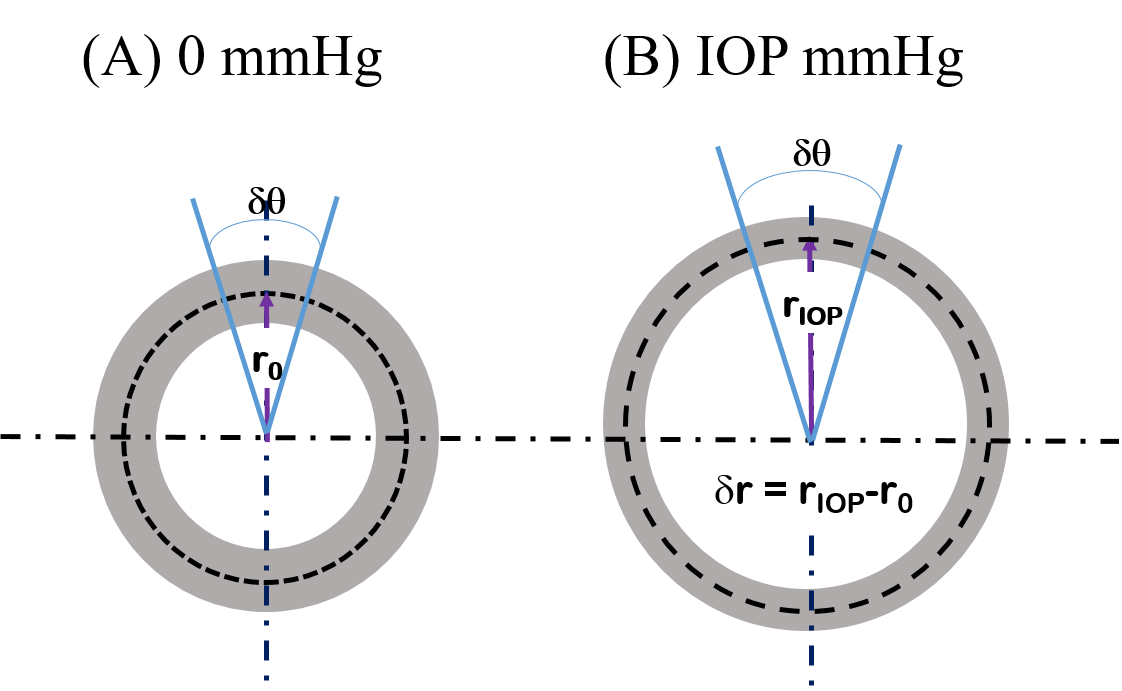


**Figure S4.** The geometrical changes of thin spherical shell under 0 mmHg (a) and IOP mmHg (b). $r$ and $\delta r$ is the mid-radius of the thin-shell and the increment of the radius accompanied by IOP, respectively.

Therefore, it is necessary to derive another formula for the strains, which is applicable to our eye phantoms. The tangential (or circumferential) strain accounts for the in-plane strain of the corneal surface of the phantom because the stress in the thin wall of the pressurized eyeball phantoms is the one of in-plane stress. An isotropic linearly elastic material subject to IOP, which is acting to the bottom of the cornea element. The element shown in Fig. S5(a) is unstrained and that shown in. Fig S5(b) is deformed by tangential stress, σ_c_. PDMS could be considered as isotropic if the deformation was small. When the cornea is inflated, as depicted in Fig. S4 and S5, the circumferential strain ε_c_ can be expressed as follows:

$\varepsilon_{c}=\frac{{L'}_{x}-l_{x}}{l_{x}}=\frac{{L'}_{y}-l_{y}}{l_{y}}$ (S4)

Assuming that $l_{x}=l_{y}$ and ${L'}_{x}={L'}_{y}$, the relative volume change $\frac{\Delta V}{V}$ can be written with Poisson’s ratio,^1^

$\frac{\Delta V}{V}= \frac{L_{x}^{' 2}\times CCT-l_{x}^{2}\times{CCT}_{0}}{l_{x}^{2}\times{CCT}_{0}}=\left( 1+\frac{L_{x}^{'}-l_{x}}{l_{x}} \right)^{1-2\nu}-1$ (S5)

If the Poisson ratio ($\nu$) of PDMS is approximately 0.4 ~0.5, the strain is less than 0.1, and the relative volume changes are less than 2.0 %. Now, we can safely suppose that

$l_{x}^{2}\times{CCT}_{0}\approx L_{x}^{' 2}\times CCT$ (S6)

By combining the Eqs. S4 and S6, the in-plane strain at the corneal apex can be expressed in terms of CCT.

$\varepsilon_{c}=\frac{{L'}_{x}-l_{x}}{l_{x}}=\frac{{L'}_{y}-l_{y}}{l_{y}}\cong\left( \frac{{CCT}_{0}}{CCT} \right)^{1/2}-1$ (S7)

The above relation between the circumferential strain and the CCT has been derived with an approximation of little but clearly occurring volume changes due to deformation. However, the error caused by the approximation could not deteriorate the accuracy of the analysis unless the strain was higher than 10 %.


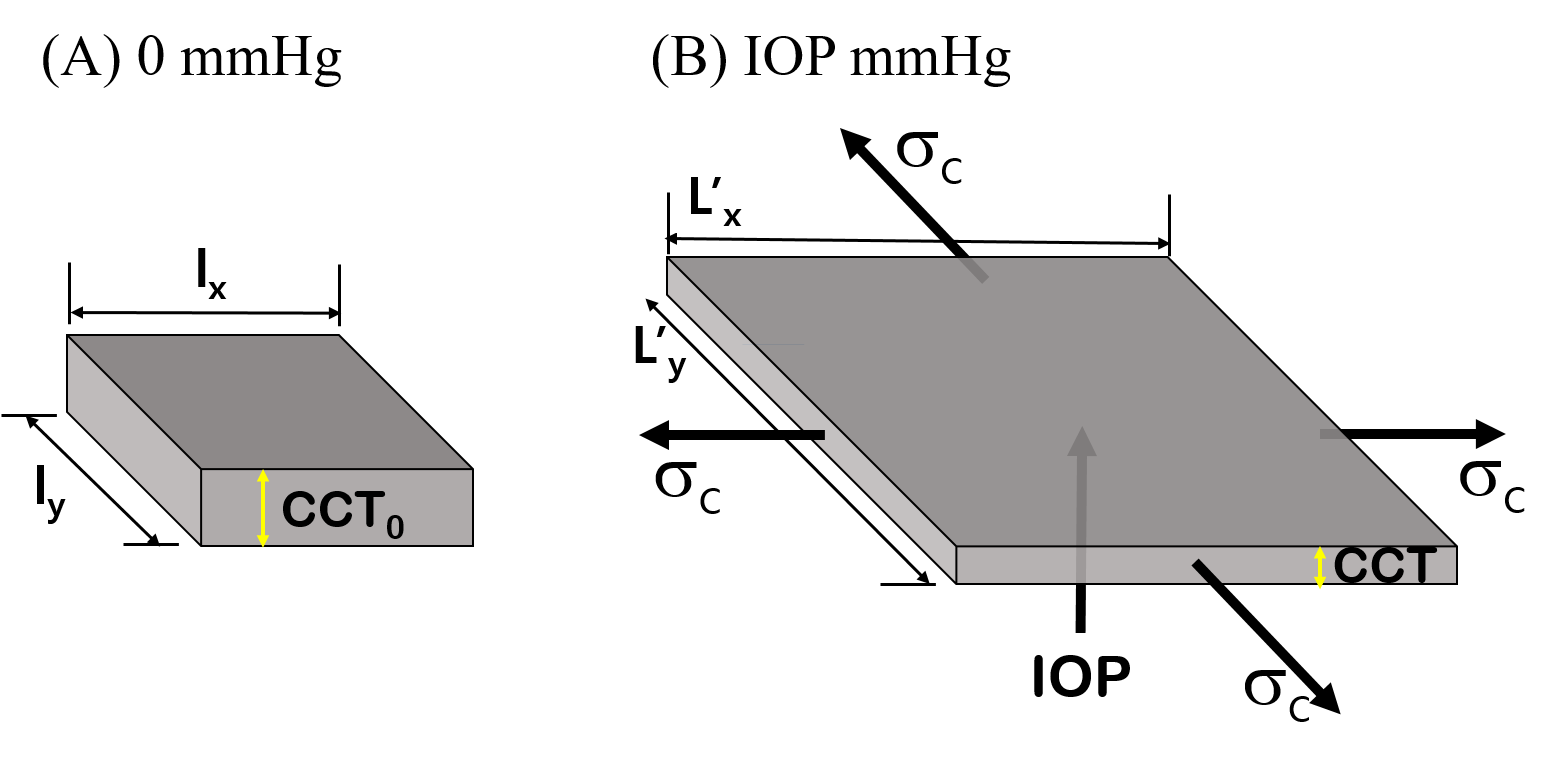


**Figure S5.** The geometrical changes of an isotropic linearly elastic material at the apex of the cornea subject to the internal pressure (IOP), which is acting to the bottom of the cornea element. The element shown in (a) is unstrained and that shown in (b) is deformed by tangential stress, σ_c_.

If the radial stress σ_r_, which is equal to the IOP, is smaller than the circumferential stress, $\sigma_{C}$, from Hooke’s law, we can describe the in-plane modulus, $\frac{E}{(1-\nu)}$

$\frac{E}{(1-\nu)}\approx\frac{\sigma_{c}}{\varepsilon_{c}}$ (S8)

where E and ν are Young’s modulus and Poisson’s ratio, respectively.

The circumferential stress-circumferential strain plot shown in Eq. S8 results in the in-plane modulus, which includes Young’s modulus and Poisson’s ratio of the cornea part of the phantoms. If then, we can discuss how the mechanical characteristics of the phantoms are similar to those of the human subject in more detail.

# 2. Supplementary references

1. Hibbeler RC. *Mechanics of Materials*. Pearson Prentice Hall; 2011.

2. Kelly, P. *Solid Mechanics Lecture notes*. Department of Engineering Science (University of Auckland: NZ, 2012).

3. Elsheikh A, Wang D, Brown M, Rama P, Campanelli M, Pye D. Assessment of Corneal Biomechanical Properties and Their Variation with Age. *Curr Eye Res*. 2007;32(1):11-19.
